# Effect of Molecular Weight of Self-Emulsifying Amphiphilic Epoxy Sizing Emulsions on the Carbon Fibres and Interfacial Properties of Their Composites

**DOI:** 10.3390/polym12112439

**Published:** 2020-10-22

**Authors:** Quantao Fang, Jiawei Yao, Kangmin Niu, Jun Tang, Yan Wei, Qipeng Guo, Chuncai Yang

**Affiliations:** 1 Jilin Institute of Chemical Technology, Research School of High Performance Fiber and Composite, Jilin 132022, China; quantaofang0712@163.com; 2 School of Chemistry, Jilin University, Jilin 130012, China; chemjtang@jlu.edu.cn; 3 School of Materials Science and Engineering, University of Science & Technology Beijing, Beijing 100083, China; sunyanmin_marie@163.com (J.Y.); niukm@ustb.edu.cn (K.N.); 4 Department of Chemistry, Tsinghua University, Beijing 100084, China; weiyen@tsinghua.edu.cn; 5 Institute for Frontier Materials, Deakin University, Locked Bag 20000, Geelong, VIC 3220, Australia; guoqipeng_1020@163.com

**Keywords:** self-emulsifying sizing emulsions, molecular weight, interfacial bonding

## Abstract

The molecular weight of self-emulsifying amphiphilic epoxy sizing emulsions has a big effect on the carbon fibres and interfacial properties of their composites. Novel amphiphilic epoxy sizing emulsions with four different molecular weights (7500, 11,000, 15,000 and 17,000) were successfully prepared by a self-emulsifying method and applied to improve interfacial bonding between carbon fibres (CFs) and an epoxy resin (EP). The effect of molecular weight on the quality of emulsions, the sized CFs and the interfacial properties of the CF/EP composite system were studied. The results reveal that these novel sizing emulsions exhibited strong emulsifying ability and high processability. The most favourable wettability and adequate CF surface free energy were obtained by the emulsion with a molecular weight of 7500. Compared with unsized CFs, the monofilament fibre tensile performance was remarkably improved when increasing the shape parameter from 5.08 to 7.20. The interfacial sheer strength (IFSS) of the CF/EP composite was greatly increased by 96% with the emulsion of 7500. The enhanced interfacial adhesion benefits were attributed mainly from the enhanced charge interaction between CFs and the sizing layer as well as the compatibility and the mechanical interlock between the sizing layer and the epoxy matrix.

## 1. Introduction

Carbon fibre reinforced plastic composites (CFRPs) are widely used in industry due to the many advantages they offer, including high specific mechanical properties, adequate protection from corrosion and fatigue resistance. The performance of CFRPs, which are composed of different materials, are affected by their components, the mechanical properties of which are dominated by carbon fibres (CFs). Conversely, the mechanical strength of CFs may be reduced due to defects and damage produced by mechanical friction in processing, transportation and use. Furthermore, the chemical inertness on CF surfaces can cause undesirable wetting of the resin matrix resulting in a diminishing effect on the quality of the interface. The interface between CFs and the resin matrix is directly related to the stress transfer efficiency from the resin matrix to CFs and the overall mechanical performance of CFRPs [1,2,3,4]. Sizing treatment can form thin films on the CF surface, which aims to fix the defects and damages resulting in an improvement of the chemical compatibility with the resin matrix. This is necessary for producing high-performance CFs in industry.

Water-based sizing has drawn increased attention recently due to its environmental friendliness in comparison to solvent-based sizing. As of now, two strategies have been developed to prepare a water-based sizing agent. The first strategy is to incorporate an emulsifier into a hydrophobic polymer to form an emulsion using a mechanical method [5,6,7,8,9]. Disadvantages of using this method include the large particle size, uneven particle distribution and relatively low stability [5,6]. Furthermore, some emulsifiers can produce poor water resistance to CFRPs [10]. The second strategy is to introduce hydrophilic groups into molecular segments of a polymer to allow it to undergo self-emulsification. Wang et al. synthesised an epoxy modified unsaturated polyester emulsion with nanoscale particle size and adequate colloidal stability via a self-emulsifying technique and used it as a sizing agent. The interlaminar shear strength (ILSS) of sized CF/unsaturated polyester resin (UPR) composites was improved [11]. In a study by Chen et al., a waterborne epoxy sizing agent for CFs was prepared by polyfunctional group novolac epoxy resin (F-51) and diethanolamine (DEOA) via a self-emulsifying technique. The modified epoxy resin had desirable water-solubility and high storage stability [12].

Thermoset epoxy (EP) is widely employed as a matrix for CFRPs because of its ideal processability and chemical stability. Epoxy-based sizing agents used for CF/EP composites were investigated in this study. To prepare a self-emulsifying epoxy sizing agent, the hydrophilic groups such as p-aminobenzoic acid (PABA) [13], diethanolamine (DEOA) [12,14], polyethylene glycol (PEG) [15] and ethanolamine (ETA) [16] need to be introduced into the molecular segment of the epoxy. In addition to the hydrophilic groups, the hydrophobic groups also require modification to enhance an epoxy in the amphiphilic epoxy sizing agent. The aim of this is to increase the compatibility between the sizing agent and the epoxy matrix.

A novel self-emulsifying amphiphilic epoxy sizing emulsion with strong emulsifying ability was developed with the purpose of improving the interfacial bonding between the CFs and the epoxy matrix. Amino-terminated polyoxypropylene (PEA) has many ether groups and terminated amino groups in its molecular chain, which implies outstanding hydrophilicity with desirable compatibility and reactivity with an epoxy. It was therefore considered an appropriate epoxy to modify for the purpose of this study. Octadecylamine (ODA) contains a long hydrocarbon chain with amino groups and can be introduced into the molecular segment of an epoxy via the opening reaction of an epoxy ring to enhance the hydrophobic properties of resulting Amphiphilic epoxy and compatibility with epoxy matrix. In this study, amphiphilic epoxy sizing emulsions with four different molecular weights were prepared by modifying an epoxy with PEA and ODA via a self-emulsifying method. The emulsions with nanoscale particle size, even distribution and strong stability were obtained. The influence of molecular weight on the emulsions, the CFs and the interfacial adhesion in the CF/EP system was investigated.

## 2. Experiment

### 2.1. Material

**Figure 1:**
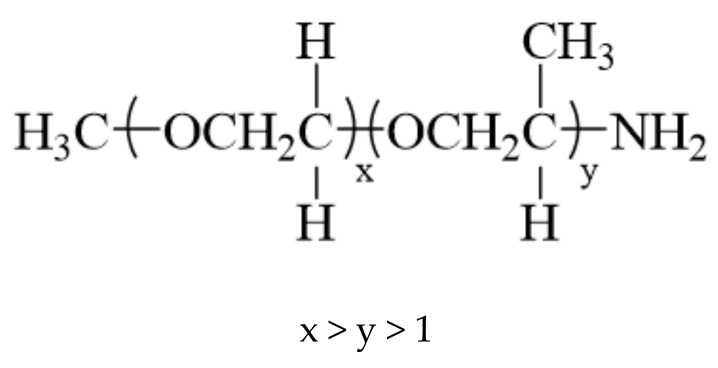
The structure of PEA.

The CFs (T700-24K) used in this study were provided by Jilin Jinggong Carbon Fiber Co. (Jilin, China), Ltd. Bisphenol A epoxy resin (E-1NT) with a molecular weight of 370 and PEA of 1000 were purchased at Jilin Qianren Innovative Materials Co. (Jilin, China) The structure of PEA is shown in Figure 1. The epoxy resin (E51) system (Hansort^®^ 6240) mixed with an amine curing agent was obtained from Tianjin Hansort Advanced Materials Co. (Tianjin, China), Ltd. The ODA was obtained from Tianjin Fuchen Chemical Reagents Co. (Tianjin, China), Ltd. Tianjin Dongfang Chemical Industry Co. (Tianjin, China) provided acetone, dichloromethane (DCM) and acetic acid. Deionised water was prepared in the laboratory.

### 2.2. Preparation of Amphiphilic Sizing Emulsions by a Self-Emulsifying Method

**Figure 2:**
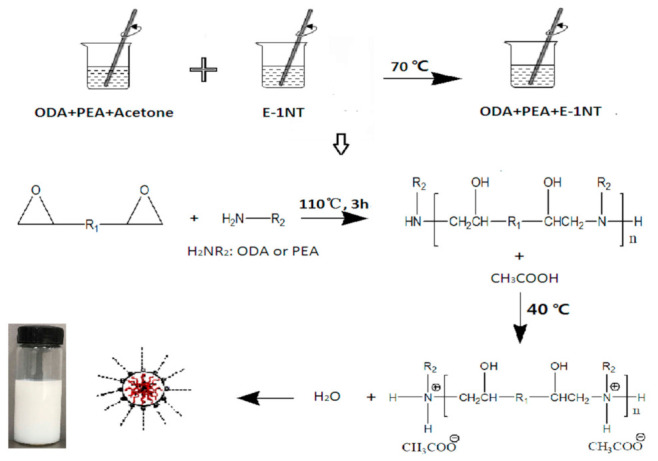
Preparation and reaction procedure of self-emulsifying amphiphilic epoxy sizing emulsions.

A schematic of the synthesising process of sizing emulsions is provided in Figure 2 [17]. ODA and PEA with a molar ratio of 19:1 [17] were dissolved using acetone, and the mixture was placed into a four-neck flask with mechanical stirrer and reflux condenser. The epoxy resin (E-1NT) was then placed into the flask in a certain proportion, as detailed in Table 1, to achieve the target molecular weight 5000, 10,000, 20,000 or 30,000 according to the theoretical calculation of Equation (1). The mixture was mixed uniformly under protection of N_2_ and maintained at 70 °C to evaporate the acetone until the temperature started to increase. Afterwards, the mixture was heated up to 110 °C for 3 h and was then cooled down to 40 °C. Finally, acetic acid and deionised water were added and a cationic emulsion with a solid content of 40% was formed. The four epoxy sizing emulsions were prepared by the same procedure but with different molecular weights. The quantities of reactants are supplied in Table 1. The notations M1, M2, M3 and M4 are used to represent the four different emulsions.

*n*_1_*M*_*ODA*_ + *n*_2_*M*_*PEA*_ + *m* (*n*_1_*M*_*ODA*_ + *n*_2_*M*_*PEA*_+ *M*_*E-1NT*_) = *M*_*polymer*_ (1)

where *n*_1_/*n*_2_ is the molar ratio of ODA to PEA and *n_1_* + *n_2_* = 1; *M_polymer_*, *M_ODA_*, *M_PEA_* and *M_E-1NT_* are, respectively, the target molecular weight of sizing agent and the molecular weights of ODA, PEA and E-1NT; and *m* is the degree of repeating units according to the *M_polymer_*.

### 2.3. Preparation of Sized CFs

The sizing emulsions were diluted by deionised water to a concentration of 3% and then put into a tank. The CF tows were passed through sizing treatment and dried immediately in an oven at 110 °C for 1 h to evaporate the water. The four types of sized CFs were prepared by the same procedure.

### 2.4. Characterisation

Gel permeation chromatography (GPC) analysis was performed using *N*,*N*-dimethylformamide (DMF) as the eluent with a flow rate of 1.0 mL/min by a GPC system (LC-20AD, Shimadzu, Tokyo, Japan). The particle size and distribution (PSD) of emulsions was tested by dynamic light scattering (DLS) (Omni, Brook, Holtsville, NY, USA). The heat resistance of sizing emulsions was examined by thermal gravimetric analysis (TGA) (TG209F1, Netsch, Exton, PA, USA). The range was 25–500 °C under N_2_ and the heating rate was 10 °C/min.

A Fourier transform infrared (FTIR) spectrometer (IR-100, Shimadzu, Tokyo, Japan) was used to measure the functional groups in the range of 4000–500 cm^−1^. Firstly, a sample was taken before the sizing agent was acidified and diluted with acetone, and then it was dried under an infrared lamp to obtain a test sample. Secondly, a background scan of the KBr sample was performed. Finally, the test sample was smeared on the KBr sample and scanned to obtain an infrared spectrum.

The dynamic contact angles (DCA) of unsized and sized CFs with water and diiodomethane (CH_2_I_2_) and surface energy were obtained by a DCA meter (DSA25, KRUSS, Hamburg, Germany). The sessile drop method was used to measure the contact angles. The unsized and sized carbon fibre tows were compacted to form a flat surface. Drops of water and CH_2_I_2_ were, respectively, dropped on the surface of carbon fibres by a syringe. The contact angle was measured by the instrument.

Scanning electron microscopy (SEM) (S-3400N, Hitachi, Tokyo, Japan) and atomic force microscopy (AFM) (Dimension Icon, Bruker, Santa Barbara, CA, USA) were used to characterise the surface morphology of sized CFs. The tapping mode of AFM with silicon tapping cantilever (OTESPA) was applied to scan the CF surface. The amplitude setpoint was 24 mV. Height images were obtained and flattened to display the CF surface clearly.

### 2.5. Monofilament Fibre Tensile Tests

**Figure 3:**
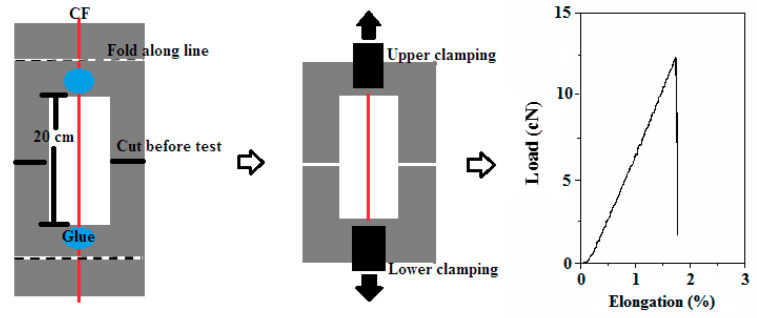
Schematic illustration of the monofilament fibre tensile testing process: sample preparation, testing procedure and load–elongation curve.

The monofilament fibre tensile tests were performed by a tensile strength tester with a force range of 100 cN (AGS-X10KN, Shimadzu, Tokyo, Japan) based on ISO 5079-1995 [18]. The tensile strength was tested with a clamp length of 20 mm and a loading speed of 5 mm/min. Glue was used to adhere the two ends of the monofilament fibre to centre the paper frame. The frame with fibre was placed in the upper and lower clamps of the tensile tester. The two borders of the frame were cut before the test. The tensile strength was deduced by the maximum load during testing. One value was tested for over 30 times. Figure 3 shows a schematic illustration of the monofilament fibre tensile testing process including sample preparation, testing procedure and a representative load–elongation curve.

Conventionally, the distribution of fibre failure is poor due to the distribution of flaws, and this is described by a weakest link model. This model allows an analysis of the monofilament fibre strength by Weibull distribution [19,20]. The statistical average intensity $\bar{\sigma }$ and the shape parameter *m* are Weibull parameters and can be deduced by using the equation of Weibull distribution (see the Appendix A). The shape parameter *m* represents the dispersion of strength data and the higher value implies fewer defects in the CF.

### 2.6. IFSS Tests of the CF/EP System

**Figure 4:**
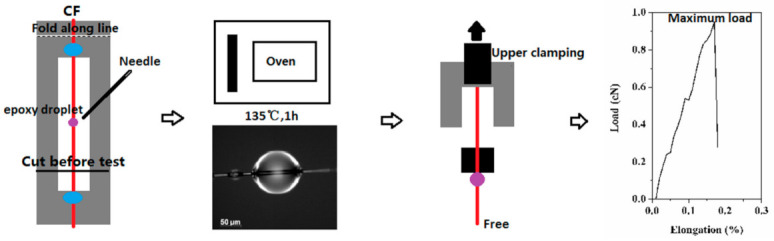
Schematic illustration of IFSS testing process: sample preparation, testing procedure and load–elongation curve.

The tests of pulling a monofilament fibre from cured epoxy droplets were performed to determine the IFSS of CF/EP composite by the tensile strength tester (XQ-1C, NFI, Shanghai, China) with a displacement rate of 1 mm/min. Glue was used to fix the CF to the paper frame. The epoxy resin (Hansort^®^ 6240, Tianjin, China) was mixed with the DCM to form the solution. The microdroplet was dripped on fibre using a needle and cured at 135 °C for 1 h. One end of the frame was placed in the upper clamp of the tester to ensure the resin microdroplet was close to the kerf. The other end was free. The IFSS value was calculated according to Equation (2). One value was obtained by testing at least five effective specimens. Figure 4 displays the schematic illustration of the IFSS testing process including sample preparation, testing procedure and representative load–elongation curve.

(2) $$IFSS=\frac{F}{\pi dl}$$

where *F* is the peak pullout force, *d* is the average diameter of the fibre and *l* is the embedded length of the resin droplet.

## 3. Results and Discussion

### 3.1. Molecular Weight

**Figure 5:**
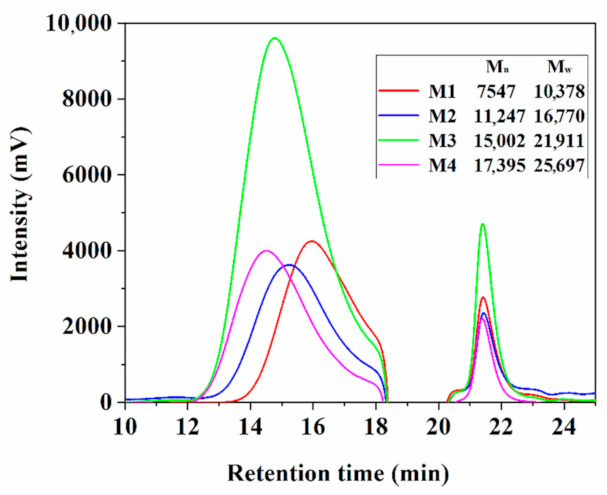
GPC results and molecular weights of four sizing emulsions.

Figure 5 displays the GPC results of four emulsions, suggesting mono-dispersity had occurred. The obtained number average (M_n_) and average molecular weights (M_w_) are also presented in Figure 5. The molecular weights of four sizing emulsions were found to be in the range of 7500–18,000 g/mol, confirming that PEA and ODA chains were grafted on the epoxy backbone. Nevertheless, the experimental molecular weight was not exactly consistent with the theoretical calculation. The three results were within the experimental error for the low molecular weight (7500, 11,000 and 15,000), but one experimental molecular weight (17,000) was smaller than the target value (30,000) because the reactants were not completely polymerised due to the high viscosity. In the remainder of this article, the new notations 7500, 11,000, 15,000 and 17,000 are used to signify the four emulsions.

### 3.2. Chemical Structure

**Figure 6:**
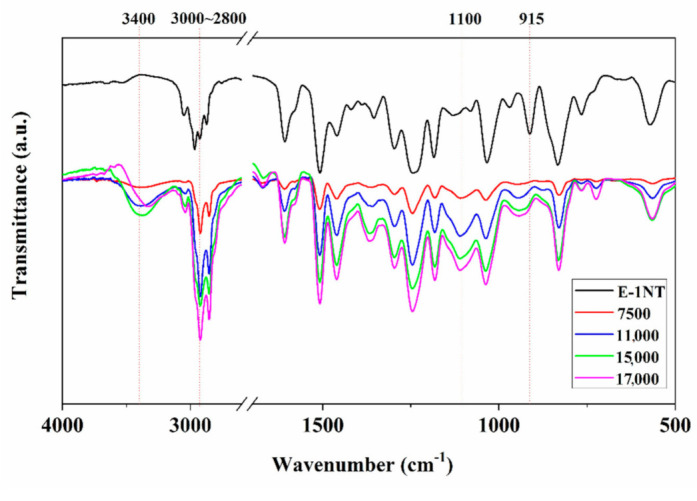
FTIR spectrogram of neat epoxy resin (E-1NT) and sizing emulsions with the four molecular weights.

FTIR results of neat epoxy (E-1NT) and all the sizing emulsions are shown in Figure 6, indicating the variation of functional groups. The peak at 915 cm^−1^ is attributed to the characteristic absorption peaks of the epoxy group, which is not visible for the synthesised emulsions. The sizing emulsions with different molecular weights exhibited similar IR spectra. A new absorption peak was observed at 3400 cm^−1^, which is assigned as the characteristic stretching vibration of hydroxyl groups. In the synthesis of emulsions, the epoxy groups underwent ring-opening reactions to form hydroxyl groups. This resulted in a decrease of epoxy groups and the increase of hydroxyl groups in the system [16]. A new wide and distinct absorption band appeared at around 1110 cm^−1^, which is characteristic of an ether group. The stretching vibration of –CH_2_ groups was found in the range of 3000–2800 cm^−1^ and became stronger as the molecular weight was increased. The spectra indicated that the emulsions were synthesised by reactions among epoxy, amino, hydrophobic and hydrophilic groups, suggesting that a successful introduction had occurred.

### 3.3. Particle Size and Distribution

**Figure 7:**
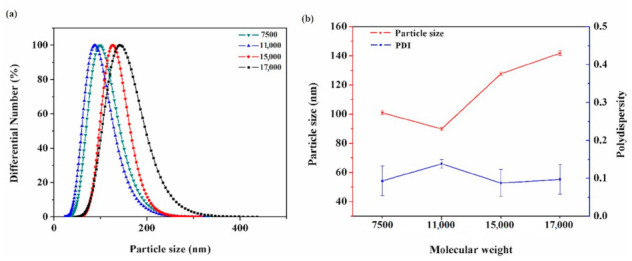
PSD of sizing agent emulsions with the four different molecular weights: (**a**) PSD test results of emulsions, (**b**) the change of particle size and PDI with molecular weight.

Figure 7 shows the PSD of the sizing emulsions with the four different molecular weights. The average particle size is stated as a nanometre grade, and these figures are 102, 90, 128 and 142 nm for the emulsions of 7500, 11,000, 15,000 and 17,000, respectively. The polydispersity index (PDI) is approximately 0.1, suggesting that the distribution was well-dispersed. The PSD results also confirm the formation of emulsion. After the addition of deionised water, the hydrophobic segments were firstly aggregated and then wrapped with the hydrophilic segments to form nanoscale particles. As a result, the particle size increased upon increasing molecular weight [21]. It is worth noting that the emulsion of 11,000 exhibited a relatively small particle size. This may be because the molecular chains of high molecular weight are prone to entanglement and the intermolecular forces become enhanced, resulting in a decrease of particle size [15,22].

### 3.4. Thermal Resistance

**Figure 8:**
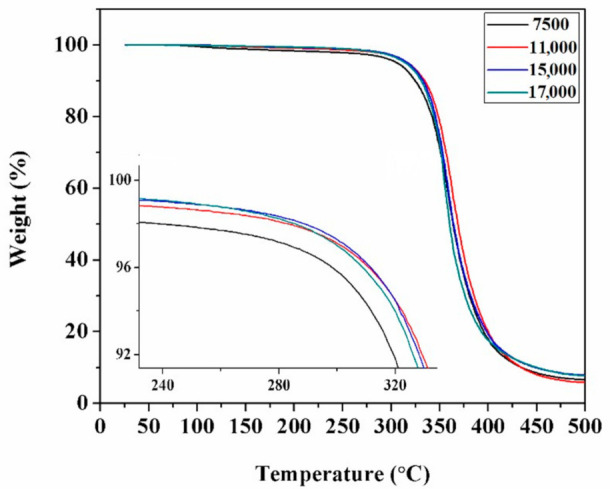
TGA diagram of sizing emulsions with the four different molecular weights.

Figure 8 represents the thermal gravimetric diagram of sizing emulsions. The emulsions showed similar thermal indications. The mass began to decrease at above 100 °C because of the presence of residual water. As the temperature was increased to 300 °C, the mass decreased dramatically. The decomposition temperatures at 5% weight loss were 306, 317, 317 and 315 °C for the molecular weights of 7500, 11,000, 15,000 and 17,000, respectively. This revealed that increasing molecular weight contributed to an improvement of heat resistance. The long molecular chain had difficulties with rotation and movement which resulted in strong thermal stability [15]. The TGA results suggest that the emulsion with the lowest molecular weight had almost the same level of heat resistance as that of the highest molecular weight. This novel type of emulsion met the requirements for the processing temperature of the CF/EP system.

### 3.5. Dynamic Contact Angle and Surface Energy

**Figure 9:**
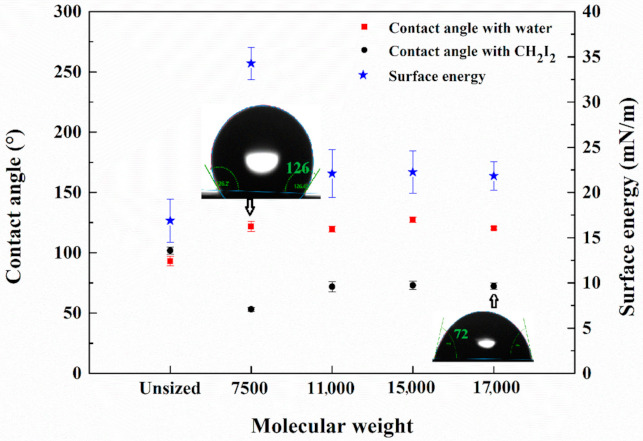
Surface energy and dynamic contact angles with water and CH_2_I_2_ of sized CFs by sizing emulsions with the four different molecular weights.

The results of DCA tests are shown in Figure 9. The contact angles with water and CH_2_I_2_ and the surface energy of unsized carbon fibres were, respectively, 93.06°, 101.86° and 16.87 mN/m. The wettability was improved by sizing procedure. The contact angles with water remained almost constant as the molecular weight changed. The emulsion of 7500 exhibited the smallest contact angle with CH_2_I_2_, resulting in the highest surface energy of 34.3 mN/m. The hydrophobic properties of ODA were probably restricted by the entanglement of molecular chains at high molecular weight [23]. To verify the speculation, acetone was added into the sizing agent of 15,000 with the weight ratio of 20% and the CFs were recoated by this sizing emulsion. Acetone is conducive to unwind ODA from the entanglement of molecular chains and allows it to self-assemble on the CF surface. DCA tests were again conducted on the recoated CFs. The dynamic contact angles with water and CH_2_I_2_ were decreased to 109.2° and 63.1°, respectively, and the corresponding surface energy was altered from 22.3 to 26.8 mN/m, confirming the speculation. DCA results showed that the emulsion of 7500 produced the best wetting effect among the four emulsions.

### 3.6. Surface Morphology

**Figure 10:**
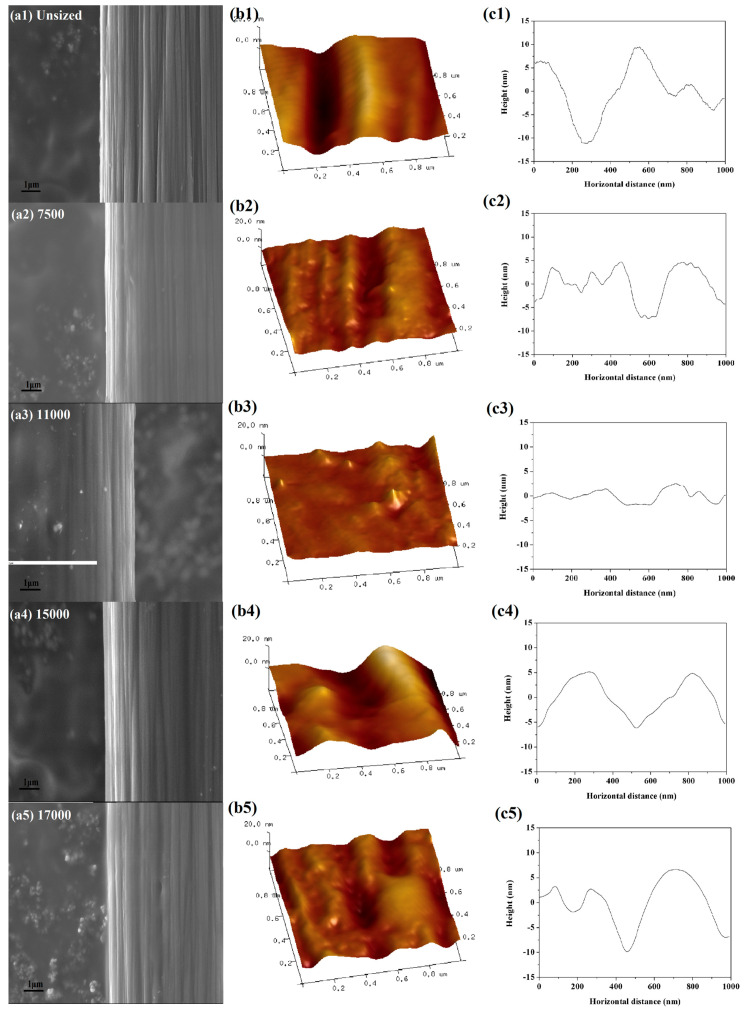
Surface morphology of CFs: (**a1**–**a5**) surface morphology by SEM of unsize CFs (**a1**) and sized CFs by emulsions of 7500 (**a2**), 11,000 (**a3**), 15,000 (**a4**) and 17,000 (**a5**); (**b1**–**b5**) 3D height images by AFM of unsize CFs (**b1**) and sized CFs by emulsions of 7500 (**b2**), 11,000 (**b3**), 15,000 (**b4**) and 17,000 (**b5**); and (**c1**–**c5**) height profiles derived from (**b1**–**b5**).

Figure 10 displays the surface morphology of CFs characterised by SEM (Figure 10a), AFM (Figure 10b) and the height profile in AFM images (Figure 10c). As shown in Figure 10(a1,b1), narrow parallel grooves were distributed on the surface of unsized CFs along the longitudinal direction. These grooves were approximately 17 nm, as shown in Figure 10(c1). The fibre surface condition was altered via the sizing process. The surface became smooth (Figure 10(a2,a3)) and the grooves were relatively shallow (Figure 10(c2,c3)). The molecular weights of 7500 and 11,000 produced excellent film-forming properties using emulsions with small particle sizes. Furthermore, using a molecular weight of 11,000 caused the grooves to be smoother after sizing (Figure 10(b3)). Nevertheless, Figure 10(a4–c5) demonstrates that some bulges were induced when the molecular weights were increased to above 15,000. This is possibly due to the emulsions with high molecular weight were not conducive to spread over CFs [24].

### 3.7. Monofilament Fibre Tensile Strength

**Figure 11:**
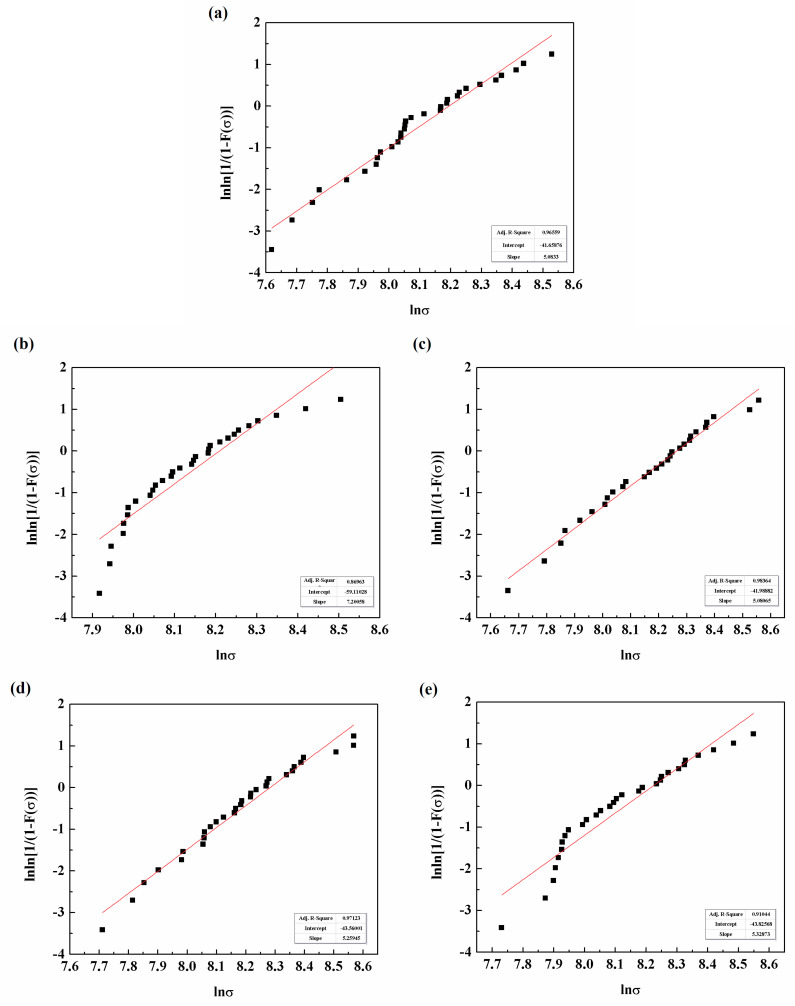
Weibull distribution of monofilament fibre tensile strength of unsized CFs and sized CFs: (**a**) unsized; (**b**) 7500; (**c**) 11,000; (**d**) 15,000; and (**e**) 17,000.

**Figure 12:**
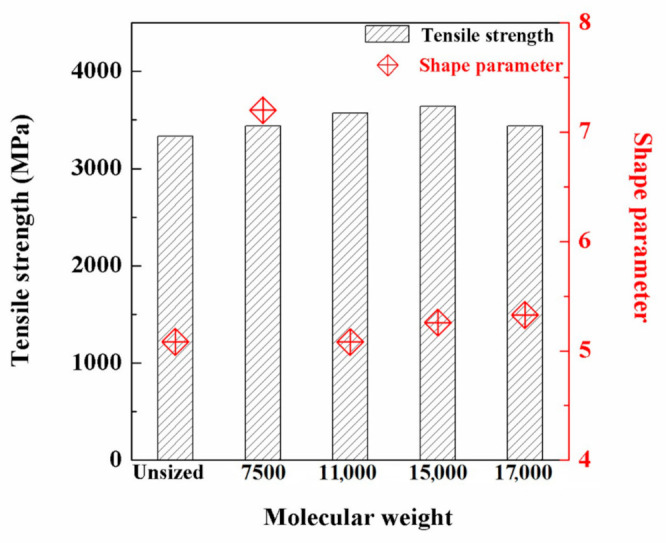
Shape parameter and tensile strength of unsized CFs and sized CFs by emulsions with the four different molecular weights.

Figure 11 displays the Weibull distribution of the monofilament fibre tensile strength of unsized and sized CFs. The corresponding slope and intercept were obtained and used to derive the Weibull parameters according to the data in the Appendix A, as shown in Figure 12. The values of the Weibull parameters were slightly different from the parameters used in previous work [17], which is due to the two different fitting methods of the basic equation of Weibull distribution. One method is called least square fitting [17] and the other is liner fitting, the latter being used in this study.

Before sizing, CFs exhibited the lowest shape parameter with a value of 5.08 due to the defects and damages on the surface. This resulted in intensive distribution of cracks and the uneven dispersion of tensile strength. The shape parameter *m* was increased for the sized CFs. The sizing emulsion of 7500 exhibited the best stability with a maximum value of 7.20, which revealed that sizing emulsions were conducive to bridge the defects and repair the damages on the CF surface in accordance with the surface morphology (Figure 10). The average tensile strength of unsized carbon fibre was 3.33 GPa, and this served as a blank control. The average tensile strengths of carbon fibre after sizing with sizing agents with molecular weights of 7500, 11,000, 15,000 and 17,000 were 3.44, 3.57, 3.64 and 3.44 GPa, respectively. It is worth noting that, generally, the larger is the shape parameter, the higher is the tensile strength; however, this relationship was not observed during this study.

### 3.8. Interfacial Shear Strength

**Figure 13:**
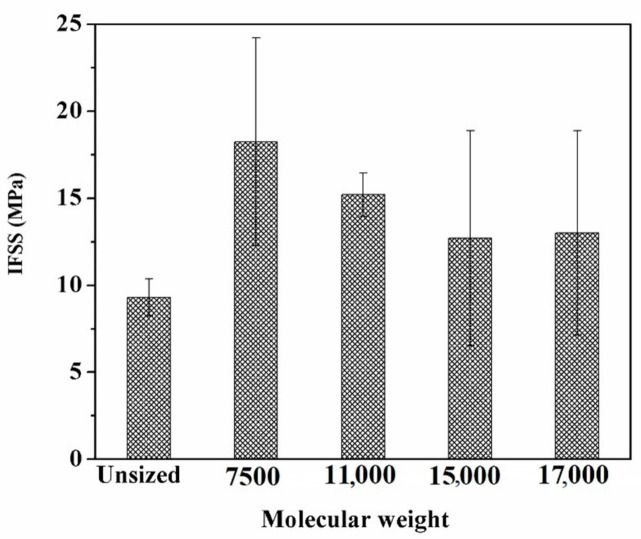
Interfacial shear strength of unsized CFs and sized CFs by emulsions with the four different molecular weights.

Figure 13 displays the IFSS results of unsized and sized CF/EP composites. IFSS of unsized CF/EP composite was only 9.30 MPa. After sizing treatment, IFSS was increased by 96.0%, 63.4%, 36.6% and 39.9% for the emulsions with the molecular weights of 7500, 11,000, 15,000 and 17,000, respectively. The descending trend with the molecular weight is displayed in Figure 13. The interfacial bonding between CFs and epoxy resin was greatly improved by the sizing process, particularly for 7500.

**Figure 14:**
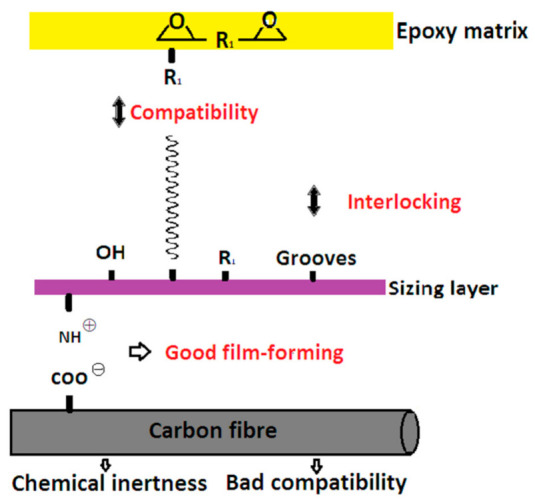
A schematic diagram of the interface enhancement mechanism between CFs and the epoxy matrix.

To illustrate the changes observed with IFSS for the sizing treatment, the interphase enhancement mechanism is sketched schematically in Figure 14. The polymer segments in the sizing agent emulsion induced by the reaction of the epoxy ring openings, such as hydroxyl and octadecyl groups and the segment of the epoxy, significantly enhanced the compatibility with the epoxy matrix and produced favourable wettability. This is beneficial to form a high-quality interface between CFs and the epoxy matrix. The sized CF/EP system by emulsion of 7500 exhibited the highest IFSS, probably due to its highest surface energy. In addition, the emulsion of 7500 maintains the fibre surface roughness without inducing irregular bulges, which contributes to the mechanical interlocking [22]. Additionally, the charge interaction between the negative CF surface and the cationic emulsion improved the film-forming property of emulsions to increase the interfacial adhesion.

## 4. Conclusions

The amphiphilic epoxy sizing emulsions with different molecular weights (i.e., 7500, 11,000, 15,000 and 17,000) were successfully prepared by a self-emulsifying method through introducing hydrophilic and hydrophobic groups into the epoxy molecular structure. The particle size of the obtained emulsions is in the nanometres range. The four emulsions prepared during this study all have strong thermal stability. When they lose 5% of their mass by heating, the required decomposition temperature is above 300 °C. The sized CFs by the emulsion of 7500 exhibited the most favourable wettability. The grooves on the CF surface became shallower when undergoing sizing treatment but the emulsions with high molecular weight (15,000 and 17,000) induced irregular bulges.

The novel sizing agent emulsion was shown to be effective for improving the mechanical performance of sized CFs and interfacial adhesion. The monofilament fibre tensile strength was enhanced by treating the defects on the CF surface. The sizing agent of low molecular weight (7500) exhibited the best quality with a tensile strength of 3.44 GPa and a maximum shape parameter of 7.20. The IFSS of CF/EP composite was enhanced by 96% with the emulsion of 7500 compared with unsized CF. This resulted from the increased charge interaction between the CF and the sizing layer as well as the compatibility and the mechanical interlock between the sizing layer and the epoxy matrix.

## Figures and Tables

**Table 1:** Quantities of reactants for sizing emulsions with four different molecular weights.

Sample	Quantities of Reactants (g)				
	ODA	PEA	E-1NT	Acetic Acid	Deionised Water
M1	100.67	19.66	127.17	22.41	404.87
M2	94.26	18.41	127.33	20.98	391.48
M3	91.42	17.85	127.73	20.35	386.03
M4	90.29	17.63	127.58	20.10	383.40

## Appendix A

The basic Weibull distribution equation is shown below

(A1) $$F\left(\sigma \right)=1-exp[-L{\left(\frac{\sigma }{\sigma_{0}}\right)}^{m}]$$

(A2) $$1-F\left(\sigma \right)=exp[-L{\left(\frac{\sigma }{\sigma_{0}}\right)}^{m}]$$

*L* is a reference length and *m* is the Weibull shape parameter. Both $\sigma_{0}$ and *m* are material constants. *F* is calculated by the below equation.

(A3) $$F=\frac{i}{N+1}$$

The strength values tested are arranged sequentially in an ascending order as $\sigma_{1}<\sigma_{2}<\cdot \cdot \cdot <\sigma_{i}<\cdot \cdot \cdot <\sigma_{N}$. *N* is the total number of samples and *i* is the sequence number of strength value.

For the Weibull distribution, the natural logarithm is taken of both sides of Equation (A2).

(A4) $$lnln\left(\frac{1}{1-F}\right)=ln\;L+m\;ln\;\sigma -m\;ln\;\sigma_{0}$$

The parameter *m* is the slop and $lnL-mln\sigma_{0}$ is the intercept of the drawn diagram. $\sigma_{0}$ can be obtained from Equation (A5).

(A5) $$\sigma_{0}=exp[\frac{ln\;L-\left(ln\;L-m\;ln\;\sigma_{0}\right)}{m}]$$

The statistical average intensity $\bar{\sigma }$ can be obtained by Equation (A6).

(A6) $$\bar{\sigma }=\sigma_{0}L^{-1/m}\Gamma (1+\frac{1}{m})$$
